# Factors influencing the participation of people with disabilities in digital skills training in Poland

**DOI:** 10.1371/journal.pone.0349514

**Published:** 2026-06-17

**Authors:** Karolina Pawłowska-Cyprysiak, Katarzyna Hildt-Ciupińska, Zofia Mockałło, Sara Hamideh Kerdar

**Affiliations:** 1 Department of Ergonomics, Central Institute for Labour Protection - National Research Institute, Warsaw, Poland; 2 The Federal Institute for Occupational Safety and Health (BAuA), Dortmund, Germany; Szkoła Główna Handlowa Warsaw School of Economics, POLAND

## Abstract

The modern world is largely built around the use of digital technologies, which are present in life. The effective use of such technologies requires appropriate competences. People with disabilities, like everyone else, require access to digital technologies to fully participate in modern life. However, considering user diversity and the accessibility of digital technologies, digital competence training can play a key role. Therefore, the aim of this article is to present the determinants of participation in digital skills training among people with disabilities in Poland. To identify these factors, a questionnaire-based study was conducted with a group of 449 people with different disabilities. Logistic regression analysis revealed that perceived availability of accessible training significantly affects participation. When training is seen as inaccessible, the likelihood of participation decreases significantly by more than 70%. These findings highlight the need to improve the accessibility of digital skills training to ensure equal opportunities for all.

## Introduction

Digital competence is defined by the European Community as the critical and confident use of digital technologies for work, leisure or communication. These competences include information and data literacy, communication and collaboration, media literacy, digital content creation (including programming), security, intellectual property issues in the digital world, digital problem solving and critical thinking. Digital competence is one of the eight key competences in the lifelong learning strand [1]. The Organisation for Economic Co-operation and Development (OECD) points out that digital competence in the modern world is as important a skill as reading, writing and numeracy [2], and its acquisition is a prerequisite for participating in, contributing to and benefiting from the global knowledge society [3]. Also, according to research, for people with disabilities, digital competence could increase opportunities for socialisation and participation in society, enhance self-confidence and help them express themselves more effectively [4] which is due, among other things, to experiencing less stigma in the online environment and less communication bias [4,5].

### Digital competences among people with disabilities

While drawing attention to the importance of digital competences in the modern world, one should not forget about groups among whom the acquisition of such competences may be difficult. This group includes people with disabilities, who may face challenges in acquiring and applying digital competencies due to insufficient accessibility, support structures, or inclusive design [6,7]. Having lower digital competences is a relevant issue especially in today’s world, as technologies are a very important component of everyday life. In this context, low digital competences can result in the emergence of digital inequalities and digital exclusion and, consequently, social exclusion. It can also deepen already existing social and health inequalities. For instance, lower digital competences may cause difficulties in understanding health apps or interpreting health information presented in such apps [– 8,9,10,11,12,11,13]. Research also shows that digital inequalities experienced by people with disabilities can impact on finding, obtaining and maintaining employment and the ability to gain promotion [14,15,13].

Several studies have found that people with disabilities often report lower levels of digital competence, which is frequently associated with barriers to access, education, or inclusive digital environments, compared to their non-disabled counterparts [16,6,17,3]. Furthermore, due to their individual needs, notable differences exist within the population of people with disabilities. Digital exclusion in this group depends to some extent on the type of disability, as well as the type and degree of needed support [18,19,2,13]. Factors such as educational level, age, and the level of required support also influence digital competence outcomes. Lower digital competence levels are more frequently reported among older adults with disabilities, people with less formal education, and individuals who experience more complex support needs [17]. Younger people are more likely to use the internet and have higher digital competencies than older people [13]. Students with disabilities often experience limited access to digital resources, support, or infrastructure—factors that can affect their academic success [20].

In their 2023 study, Lazić et al. (2023) [21]  examined digital competencies and self-assessments, finding that people with disabilities rated their general skills, information and data literacy, communication and collaboration abilities, digital content creation, cybersecurity awareness, and technical problem-solving skills highly. However, studies by [5,22,23] showed that while people with visual impairments rated their digital competencies highly in self-assessments, external evaluations conducted during task-based computer activities indicated significantly lower actual competence levels. Despite the widespread digitization of contemporary societies and increasing research interest in this area, the literature on the digital competencies of people with disabilities remains limited. Existing studies predominantly focus on subjective self-assessments rather than objective evaluations. Moreover, little attention has been given to the factors influencing the participation of people with disabilities in digital skills training programs.

### Digital competence training as a form of bridging the digital divide

Bridging the digital divide—or addressing digital exclusion—among people with disabilities requires a multifaceted approach. For instance, focusing on the lived experiences of people with visual impairments and blindness, a recent literature review underscored the critical importance of accessible digital technologies for this group [24]. Despite ongoing efforts to improve accessibility, such as the development of guidelines and support measures, research indicates that many people with disabilities benefit significantly from training tailored to their needs [6,24]. While some users manage to develop personal strategies to navigate or circumvent inaccessible platforms, structured training can enhance both their competence in using assistive technologies and their overall digital literacy. In their study on digital competency among university students, Cabero-Almenara et al. [25] concluded that training programs should be universally accessible and available to all students, designed with universal accessibility in mind to ensure inclusivity for all learners, including those with diverse support needs. In this regard, the study by Gunupudi et al. [26] found that individual-based training of mobile digital skills for people with sensory impairments could be helpful in addressing their specific access needs. In this context, training in digital competencies holds considerable potential to help people with disabilities more effectively engage with the digital environment in social, educational, and cultural spheres [7,8,27]. Moreover, these training initiatives could facilitate access to digital employment and promote greater equity in labour market participation for people with disabilities [28,29].

Considering the central role of digital competencies in both social participation and employment, and the critical importance of digital competence training, this study aims to examine the digital skills of people with disabilities in Poland, with a particular focus on how they self-assess their digital capabilities and experiences. It explores the role and perceived impact of digital competence training by comparing individuals who have participated in such training with those who have not. Furthermore, the study seeks to identify key factors that influence the willingness of people with disabilities to engage in digital training initiatives. By addressing these dimensions, the research contributes to a deeper understanding of the barriers and enablers of digital inclusion, and offers insights into how inclusive and tailored interventions can support the development of digital skills, ultimately enhancing access to digital participation and employment opportunities for people with disabilities.

## Materials and methods

A cross-sectional, anonymous questionnaire survey was conducted with a group of people with disabilities in order to achieve the stated aim. Participants with disabilities were recruited for the study through foundations, organizations, and community groups supporting people with diverse disabilities (mainly clubs and associations), as well as via online forums and social media platforms. A purposive sampling strategy was employed, focusing specifically on individuals with physical, visual, or hearing disabilities. Additionally, a snowball sampling method was used to expand the participant pool through referrals. A purposive sampling approach was chosen because the study’s focus was on a clearly defined population limited to the three aforementioned types of disabilities. Although this approach limits representativeness, it allows for a more focused examination of groups that are particularly relevant to the study’s objectives.

The study group was divided into two equally sized subgroups based on participation in digital skills training. Group assignment was determined by responses to a screening question: “Are you currently participating in any training aimed at improving your digital competencies?” The recruitment process was designed to ensure that 50% of the participants were women and 50% were employed. Participants were required to have one of three types of disability (motor, visual, or hearing impairments). The sample was divided by gender and employment status based on participants’ self-reported answers. The two questions asked were: “Please indicate your gender” and “Are you currently employed”? To assess the level of disability, participants’ self-reported responses to a closed-ended question “What level of disability do you have?” were used (respondents could indicate one of three levels of disability severity: mild, moderate, or severe). Additionally, participants were drawn from both urban and rural areas in Poland to reflect a diverse range of living environments.

The exclusion criteria included the absence of a disability, the presence of a disability other than motor, visual, or hearing impairment, and age below 18 years.

### Ethical statement

The implementers of the study were granted consent by the Bioethics Committee at the Institute of Rural Medicine in Lublin, Poland (resolution number 16/2023). The scientific study was conducted in accordance with Regulation (EU) 2016/679 of the European Parliament and of the Council of 27 April 2016 on the protection of natural persons with regard to the processing of personal data and on the free movement of such data and repealing Directive 95/46/EC (General Data Protection Regulation).

The study was conducted from July to September 2024. The participation in the study was preceded by the participant’s written informed consent to take part in the study.

### Research tools

To address the research questions, both self-developed and standardized questionnaires were utilized. While a detailed description of the research instruments can be found in S1 File, an overview of the instruments used in this study is provided below.

Firstly, a questionnaire was designed solely for the purpose of the study. The original survey was developed based on a comprehensive review of relevant literature, research reports, and European guidelines concerning digital competencies.

Additionally, the following standardized tools were used:

- Work Ability Index (WAI) by Tuomi, Ilmarinen, Jahkola, Katajarinne, Tulkki, in Polish translation by [30]– a questionnaire assessing subjectively perceived ability to perform work.
- Copenhagen Psychosocial Questionnaire (COPSOQ II) by Kristensen, Hannerz, Hogh and Borg, 2005 [31], Polish adaptation by Baki Ł. - includes a self-efficacy subscale that describes generalized beliefs of participants that, regardless of circumstances, they are able to cope with difficult problems, unexpected situations, and realize their own plans and intentions.
- Rosenberg’s Self-Esteem Scale (SES), Polish adaptation by [32]– a scale assessing general self-esteem, understood as a relatively stable disposition reflecting a positive or negative conscious attitude towards the Self.
- Multidimensional Scale of Perceived Social Support (MSPSS) by G. Zimet, N. Dahlem, S. Zimet & G. Farley, Polish adaptation by [33]– this scale captures the multidimensionality of perceived social support, recognizing three primary sources of support: a significant person, family, and friends.

A detailed description of the research instruments is provided in Supplementary file - S1 File.

Before completing the paper questionnaires, all participants were fully informed about the purpose of the study, the confidentiality of their data, and provided written informed consent to participate. The process of obtaining informed consent, including providing study information and collecting consent, was adapted to meet the participants’ needs. An example of such an adaptation was the possibility of providing informed consent in electronic form using a form developed in accordance with WCAG 2.1 standards.

### Statistical analysis

The data were analyzed using IBM SPSS Statistics 29. The analysis included:

- descriptive statistics to characterize the study group,
- calculation of the Cronbach’s α coefficient to assess the reliability of the standardized questionnaires used with the participants,
- Mann-Whitney U test to evaluate the significance of differences between groups for continuous variables,
- Chi-square test to assess the significance of differences between groups for categorical and nominal variables,
- The Variance Inflation Factor (VIF) test was used to assess the presence of multicollinearity among the predictors;
- logistic regression analysis to identify factors predicting participation in digital competence training among people with disabilities.

## Results

### Demographic information

A total of 449 participants with disabilities took part in the study. The average age of the study group was 41 years (min = 18, max = 66, SD = 14). The largest number of people lived in a medium-sized city, from 20 to 100 thousand inhabitants (38%), the largest group were those with basic vocational education (27%), while those with lower secondary education constituted the smallest group (1%). Among the employed, 80% worked in the private sector, of which 62% in the open labour market.

Considering the data on disability, 48% of the group had a moderate degree of disability, 38% a mild degree and 14% a severe degree. The largest percentage of respondents (59%) had a motor disability, 23% had a visual disability and 18% a hearing disability. The average duration of living with a disability was 16.5 years (min = 1, max = 65, SD = 13.5).

### Digital competences

The study’s participants defined digital competences as general skills for using specific digital tools and new technologies, using technologies or finding themselves in the digital world. One person pointed out a more general answer: *“I recognise that digital competences are a harmonious composition of knowledge, skills and attitudes enabling life, learning and work in a digital society, i.e., a society using digital technologies in everyday life and work.”*

Among the responses, some indicated limited familiarity with the concept of digital competences, describing them as telephone support, basic computer operation or only the ability to turn on a device. 18% of the total group indicated that they do not know what digital competences are. One participant indicated a lack of interest in this area.

96% of the study group use new technologies in work and life, most often at home (86%), 43% use them at work. 4% of respondents indicated that they are using new technologies in other situations, for example, in science, studies, travel, or leisure time. On a daily basis, 56% of the group studied use assistive technologies.

People with disabilities most often use digital competences when establishing or maintaining relationships, using electronic banking, searching for information or shopping online. 70% of the group considered that contemporary digital competences are important competences of the employee, and 47% of the group pointed out that workers, depending on the profile of the company in which they are employed, should have advanced digital competences. More than half of respondents (52%) are interested in having advanced digital competences.

### Self-assessment of digital competences

The largest group of surveyed people with disabilities rated their level of digital competence as average (49%), 26% as low, 18% as high. The lack of competence was indicated by 5% of respondents, while 2% indicated a very high level of digital competence. They also rated their skills in using computers, MS Office or similar software, and web browsers as moderate (Table 1).

**Table 1:** Distribution of responses to questions about the degree of knowledge of computer use, MS Office or similar package and web browsers (%, N = 449).

	Using a computer	Using MS Office or similar	Using internet browser
Low	32%	40%	28%
Average	52%	46%	48%
High	16%	14%	24%

Participants were presented with a list of 27 digital competences and asked to assess their own level of proficiency on a scale from 1 – no competences to 5 – very high competences. Competences such as sending and receiving e-mails and saving photos, documents, etc. were rated as very high. In contrast, programming and virtual team management were the areas in which participants reported the lowest competence levels (detailed data about the distribution of answers of respondents regarding self-assessment of selected 27 digital competences are presented in Supplementary file - S1 Table).

Participants were asked whether they could identify what gaps they have in the field of digital competence. 35% answered “rather yes”, 27% “do not know”, 19% “rather not”, 10% “definitely yes”, and 9% “definitely not”. The areas most commonly identified for improvement included programming, website development, Excel use, AI tools, data analysis, application development, automation and management, cybersecurity, and general digital literacy.

### Digital competence training

According to the study assumptions 50% of participants were currently receiving digital competence training, 10% had participated in such training in the past, 10% were not currently taking part in any training, and 30% had never received digital competence training. Among those who were currently or previously involved in digital competency training, 58% of participants indicated that they attended it on their own.

Among participants currently receiving digital competence training, the largest group consisted of individuals with motor disabilities (59%), 22% had visual disabilities and 19% had hearing disabilities. Similarly, among those not currently participating in training, the majority were individuals with motor disabilities (59%), 24% had visual disabilities and 17% had hearing disabilities.

The most common motivation for participating in digital competence training was the possibility of upgrading, retraining or acquiring new qualifications (61%), the least frequently motivated by encouragement from employers or colleagues (13%).

Among participants who were not currently engaged in digital competence training, the most frequent reason for this was not needing digital skills in daily life (32%) or lack of funding (30%), the least frequent reason was lack of support from employers or colleagues (1%) or other reasons such as lack of computer hardware (2%).

69% of respondents stated that current digital competency training is being conducted appropriately. The organization of the training was most often assessed as good (49%). When asked about the availability of digital competence training for people with disabilities, 30% of respondents indicated that they are available, 23% indicated that only some elements of this training are available, 14% indicated “I don’t know”, and 10% said the training was not available. The remaining 23% indicated that the subject of availability of training was not relevant to them. As the most commonly cited barriers, participants identified the place where training was organised and its forms (33% each), training materials (27%), training content or training in general (23% each), and 1% were unsure which elements were inaccessible. 4% of respondents indicated a different answer (e.g., difficult language, training costs, training hours).

As the most effective form of digital competency training, participants with disabilities identified stationary lectures combined with problem solving, tasks – workshops (42%). Participants currently or previously involved in digital competence training reported the training was effective (64%) and that thanks to this training their skills improved to a very large extent (38%). Participants reported using these skills most often in everyday life (58%), at work (44%), to retrain and change jobs (18%), for promotion or personal interest (11% each).

43% of the respondents expressed interest in participating in digital competency courses in the future, 41% were unsure, and 16% were not interested in such courses. According to participants with disabilities, topics which should be addressed at these courses are cybersecurity, programming, automation, the use of artificial intelligence, internet marketing, cloud presentations, the creation of websites according to WCAG guidelines, and geoinformatics. Among the answers were also such as basic computer skills, psychological support, and reducing barriers such as anxiety related to new technologies.

### People participating in training vs. non-trainees

To identify potential differences between people with disabilities who participated in digital competence training and those who did not, statistical analyses were conducted — the Mann-Whitney U test was used for continuous variables and the Chi-square test for nominal and categorical variables. General assessments of digital competence and specific digital skills (e.g., use of web browsers, text editing) were not included in this table, as in all of these areas, participants in digital competence training demonstrated higher levels of competence. This outcome is likely related to their participation in the training programs.

When analysing the results, several differences were observed between people with disabilities who participated in digital competence training and those who did not. Participants in training tended to:

- be younger,
- more often have a slight degree of disability,
- better assess their current ability to work compared to the lifetime best,
- indicate greater mental resources,
- perceive higher level of social support (both general and from key individuals, such as special person, family or friends),
- express a stronger sense of self-efficacy and self-esteem,
- be more often employed,
- have more frequently participated in other training or educational courses,
- more often report that digital competences are required in their work,
- use new technologies and assistive technologies more frequently in daily life,
- more often consider digital competences to be important for employees,
- more often indicate that workers should have advanced digital competences,
- express greater interest in acquiring advanced digital competences,
- be more aware of their own digital competence gaps,
- report that their employers support or manage access to digital competence training,
- be more likely to perceive digital competence training as available to people with disabilities, and express stronger interest in taking part in such training in the future.

Detailed results, including percentage distributions and the results of the Mann–Whitney U test and the chi-square test, are presented in Supplementary file - S2 Table.

### Factors associated with the participation in digital competence training among people with disabilities

To explore the factors associated with participation in digital competence training among people with disabilities, a logistic regression analysis was conducted. This analysis allowed for the identification of variables linked to a greater likelihood of participating in such training, as well as those associated with a lower likelihood of participation.

The dependent variable was the dichotomous variable *“participation in training in the field of digital competences”*. The reference category was coded as 1 to indicate current participation in digital competence training*,* while the value 0 indicated non-participation.

According to the methodological assumptions, the analysis included variables measured on the quantitative, ordinal and nominal scale. The model included the following variables: age, gender, place of residence, level of education, degree of disability, type of disability, duration of disability, everyday use of new technologies, everyday use of assistive technologies, belief in the importance of advanced digital competences for modern employees, interest in developing such competences, belief in the accessibility of digital competence training for people with disabilities, willingness to use digital competence training in the future, assessment of self-reported digital competences, assessment of current ability to work compared to the best ability to work in life (question 1 from WAI), mental resources (WAI), self-assessment (SES), social support (MSPSS), self-efficacy (COPSOQ). Factors that were assessed in both employed and unemployed participants were selected for inclusion in the model.

To evaluate multicollinearity among the variables, the Variance Inflation Factor (VIF) was calculated. The analysis yielded VIF values ranging from 1.097 to 2.277, indicating moderate multicollinearity, which is generally considered acceptable. Detailed VIF results are presented in Supplementary file - S3 Table.

The optimal model built using explanatory variables includes 3 predictors:

- interest in acquiring advanced digital competences,
- self-assessment of digital competences,
- perceived availability of digital competence training for individuals with disabilities (Table 2).

**Table 2:** Factors significantly associated with participation in digital competence training among individuals with disabilities (results of logistic regression analysis).

	B	Standard Error	Wald	Df	Significance	OR
**Interest in having advanced digital skills (no interest vs. interest)**	−0,488	0,231	4,450	1	0,035	0,614
**Assessment of own digital competences (low vs. high rating)**	0,597	,0255	5,483	1	0,019	1,817
**Assessment of the availability of digital competence training for people with disabilities (training is available vs. training is not available)**	−1,331	0,148	81,245	1	<0,001	0,264
**Conviction of the need for modern employees to have digital competences at an advanced level (belief that the employee should have such competences vs. belief that he should not have such competences)**	−0,506	0,279	3,301	1	0,069	0,603

At the level of statistical trend, participation in digital competence training was also influenced by the belief that modern employees are expected to possess advanced digital competences (Table 2).

In accordance with the assumptions of the statistical procedure, the final model included variables that demonstrated the strongest association with the dependent variable. The criterion for inclusion in the model was their statistical significance.

This model explains about 70% of the variance of the dependent variable (Table 3).

**Table 3:** Summary of the model of participation in training in the field of digital competences of people with disabilities.

− 2 logarithm of credibility	Cox’s R-square and Snell’s	Nagelkerke’s R-square
283,645	0,529	0,705

Based on the odds ratio, it can be concluded that:

- Lack of interest in acquiring advanced digital competences reduces the likelihood of participating in digital competence training by 38% (OR = 0.614),
- Perceiving digital competence training as inaccessible to people with disabilities reduces the likelihood of participation by 74% (OR = 0.264),
- Not being convinced that modern employees should have advanced digital competences reduces the likelihood of participation by 39% (OR = 0.603),
- A higher self-assessment of one’s digital competences nearly doubles the likelihood of participating in such training (OR = 1.817).

## Discussion

In today’s world, digital technologies increasingly shape how people work, spend their leisure time, and build social relationships. For people with disabilities, these technologies offer significant opportunities to enhance quality of life, foster social inclusion, and support greater independence and access to information and services [34,35,36,37,25]. However, due to many factors such as inaccessibility of technologies, having a disability could also be associated with barriers to technology use [38,39]. These challenges may lead to lower levels of digital competence and, in turn, contribute to social exclusion, digital isolation, and the persistence of digital inequalities [8,9,10,40], resulting in deeper social divisions and unequal life opportunities [10]. Nevertheless, literature shows that people with disabilities actively use digital technologies to work, maintain relationships, manage finances, shop online, and search for information—activities that foster greater autonomy [5,41,42,5]. Although comprehensive global data on the digital skills of people with disabilities is still limited, literature suggests that their competence levels are generally lower than those of persons without disabilities [16,4], and that this digital divide begins early—in school education [43] impacting later success in higher education as well [20]. In this regard, respondents with disabilities which were participants in the study often described their digital skills as average, which aligns with findings by Cabero-Almenara et al. [25], who observed similar self-assessments among students with disabilities in Chile, with a notable portion unable to clearly define what digital competence entails. Digital competencies are therefore critically important — especially for persons with disabilities — and it is essential to conduct broad and inclusive research in this area to support the acquisition and development of these skills within this population.

### Education for digital competencies: Insights from research findings

The result that only half of the group is interested in having advanced digital competencies, and an even smaller percentage of respondents want to develop their digital competencies in the future, is disturbing. Taking into account the general trends in the development of their own competencies in Poland in 2017–2021, only 55% of people with disabilities have developed their competencies in any way in the last year preceding the survey, compared to 81% of people without disabilities. Poor health and various types of limitations have a significant impact on opportunities for broad-based development [44,45,17].

Logistic regression analysis showed that participation in digital competency training for people with disabilities depends on 3 factors: interest in having digital competencies at an advanced level, evaluation of one’s own digital competencies, and evaluation of the availability of digital competency training for people with disabilities. At the level of statistical trend, participation in digital competence training is also influenced by the belief that today’s employees need to have digital competence at an advanced level. Additionally, one important element related to the availability of digital competence training indicated by respondents is its affordability. This result confirms the literature review by Wright et al. [40], which indicates that the use of digital tools depends, among other things, precisely on the cost they entail. Easy access to education and its low cost are two factors that determine the motivation to undertake training in general (in the general population) and influence the willingness to undertake lifelong learning [46]. Many people with disabilities face economic challenges that can limit their access to digital technologies and related opportunities [47,6,5]. Limited financial resources may lead to prioritizing essential needs over investment in new technologies [48].

The accessibility of digital competence training for people with disabilities is a very important aspect. As shown by the results of this study, the perception that such training is inaccessible reduces participation rates by more than 70%. Therefore, it is important to design accessible training, including accessible training materials, to improve the digital skills of people with disabilities and enable their participation, for example in employment [15]. As visual or musculoskeletal impairments may restrict access to digital technologies [49,11], the adoption of a universal design approach is recommended. The issue of accessibility is a contemporary topic of discussion and action for people with disabilities. In Poland, on 26 April 2024, the Sejm passed the Act ‘On Ensuring that Business Entities Meet the Accessibility Requirements for Certain Products and Services’, which transposed an EU directive into Polish law, called the European Accessibility Act (EAA) [50]. Its aim is to facilitate access to products and services for as many people as possible, regardless of ability or special needs, and to raise public awareness of accessibility. Lack of access to digital content (whether physical or lack of skills to use such media) can negatively affect social inclusion, education and work, and overall quality of life [5].

It may seem surprising that a better assessment of one’s digital competence increases the likelihood of participating in digital training. Such training improves competence and enables the identification of any gaps in knowledge or skills. Furthermore, if its effectiveness is recognised, it influences one’s decision to continue training in order to further improve one’s competence. This is consistent with existing literature on the motivation to undertake training. It indicates that previous learning experiences influence the take-up of further learning. Positive experiences increase motivation for further development and can also enhance self-confidence and shape long-term readiness to upgrade skills [51,52,53,54].

The results of the current study indicate that participants reported anxieties of new technologies, particularly a lack of confidence in using the internet and digital tools responsibly. One participant explicitly expressed a desire to overcome this fear, stating: “...how not to be afraid of the Internet and online networks, and how to use the Internet in a responsible manner.” Therefore, it is particularly important to address this topic in relation to teaching new technologies. This is in accordance with the results of a report by the Orange Foundation [55] and the Consumer Federation (2021) [56] from Poland. Digital exclusion is more related to attitudes towards new technologies and towards one’s own abilities and skills than to a lack of physical access to them. To a greater extent, this correlation is noticeable among people with disabilities. Fear, lack of confidence and safety concerns are identified by the literature as barriers to accessing digital technologies [49,5,11]. They talk about so-called digital attitude, i.e., willingness to use digital resources, the attitudes towards technology, the benefits it brings and the risks it may cause. They are determining factors in how patterns of both digital inclusion and exclusion are shaped. Through digital attitudes, it is also possible to understand digital skills and digital access disparities between different populations [57,58,11,59].

It is essential to promote comprehensive education in the field of digital competences, including their understanding, practical application, and its role in contemporary society. Such education should aim to reduce knowledge disparities among people with disabilities, which become particularly evident when individuals are asked to define digital competences. Although some people with disabilities demonstrate an understanding of the importance and complexity of digital competences, a considerable proportion report limited knowledge or define them at a very basic level. Special attention should be given to the fact that digital competences currently constitute essential skills for employees—both for gaining and maintaining employment.

### Implications for social policy

Bridging emerging digital inequalities and reducing digital exclusion of people with disabilities requires a number of measures, including training, workshops or courses to facilitate the use of new technologies [8]. In the present research, the study group was divided by design so that 50% were people currently participating in such trainings The most common reason for undertaking training in this group was the opportunity to improve skills, retrain, or acquire new qualifications, whereas non-participation was mainly attributed to perceived limited relevance or financial constraints. These findings are consistent with research conducted by the Consumer Federation (2021) [56], which identifies these factors as key contributors to digital exclusion. Also, data from the Human Capital Survey conducted in Poland indicate that the excessive cost of training is a barrier to participation and thus to developing one’s own capabilities [17]. The impact of financial capability on not taking up digital education is also indicated by the results of Tarrayo et al. [60]. According to this research, the lack of adequate financial resources alongside insufficient personalization of the curriculum, and in addition to manifestations of discrimination and stereotyping, are the three main factors determining non-take-up of digital literacy education.

There is also a clear need for psychological support to help people with disabilities overcome their fears related to new technologies, encourage an open attitude towards technological innovation, and highlight the opportunities for learning how to use digital tools. Fear of new technologies is not exclusively expressed by people with disabilities; it has also been identified by employers, as well as the representatives of training institutions that provide digital skills education for people with disabilities. Such fear and anxiety can pose significant barriers to participation in training activities, often stemming from a perceived inability to learn how to use digital technologies, resulting in a reduced competence in this area.

### Directions for future research in the field of digital competences among people with disabilities

Research on digital competencies and their development among people with disabilities — especially in Poland — is highly important. Today, these skills largely determine effective functioning in professional and social environments. The presented study highlights directions for actions needed to support the development of digital competencies among people with disabilities and identifies key considerations for fostering these skills inclusively.

A limitation of this research is its subjective nature. Participants assessed both their own competencies and the trainings, including their accessibility, based on personal perspectives and experiences. This may lead to biases in the responses provided. Another limitation is that the study involved only individuals with three types of disabilities. It would be important to explore the perceptions of people with other types of disabilities as well. Additionally, the range of factors influencing participation in digital skills training should be expanded.

From the perspective of future research on the digital competences of people with disabilities, several priorities can be identified for further investigation. First, the research methodology could be strengthened through the inclusion of qualitative approaches—particularly in-depth, face-to-face interviews—which would complement questionnaire-based studies and allow for a more nuanced interpretation of quantitative findings. In addition, future studies should incorporate factors not addressed in the present research, such as a more comprehensive analysis of personality-related variables and a broader examination of social and environmental influences. Expanding the research sample to include individuals with other types of disabilities would also enhance the generalizability of the findings. Finally, conducting research among employers and institutions that provide digital competence training, as well as considering the inclusion of close family members, would introduce valuable additional perspectives and contribute to a more holistic understanding of the digital competences of people with disabilities.

## Supporting information

S1 File Supporting information. A detailed description of the research instruments used.
(DOCX)

S1 Table Distribution of answers of respondents regarding self-assessment of selected 27 digital competences (in %, N = 449). (DOCX)

S2 Table Characteristics of participants with disabilities who did and did not take part in digital competence training – Results of Mann-Whitney U test and Chi-square test (N = 449). (DOCX)

S3 Table VIF test results (Variance Inflation Factor). (DOCX)

S1 Dataset Anonymized dataset underlying the findings. (XLSX)
