# Supplementary material for: Factors influencing the participation of people with disabilities in digital skills training in Poland
Source: PLoS One. 2026 Jun 17;21(6):e0349514. doi: 10.1371/journal.pone.0349514 (PMC13274863; doi:10.1371/journal.pone.0349514)
Supplement: S1 File — A detailed description of the research instruments used. (DOCX) [file pone.0349514.s001.docx]

Supplementary files

**S1 Supporting Information.** A detailed description of the research instruments used.

Questionnaire designed solely for the purpose of the study - It comprised 47 questions, organized into the following thematic sections: a) demographic information (including age, gender identity, place of residence, educational attainment, and disability characteristics); b) employment information (including current workplace, job role, and the extent to which digital competencies are required in the professional context); c) digital competencies (including participants’ understanding of the concept, self-assessment of their digital skills, and the application of these competencies in daily activities; the areas of digital competence included in the survey were based on the Digital Competence Framework for Citizens (DigComp 2.2) (Vuorikari; Kluzer; Punie, 2022). DigComp focuses on eight proficiency levels and examples of use, and on an analysis of European Union sources); d) training in digital competencies (covering motivations for participating in training, barriers to access, evaluation of completed training programs, perceptions of training availability and accessibility, assessment of training formats and materials, and future intentions regarding digital upskilling). Of the 47 items, 32 were single-choice questions (6 of which included an “Other” option with space for elaboration), 9 were multiple-choice questions (all with an “Other” option and an open response field), and 7 were open-ended questions.

Additionally, the following standardized tools were used:

• Work Ability Index (WAI) by Tuomi, Ilmarinen, Jahkola, Katajarinne, Tulkki, in Polish translation by J. Pokorski — A questionnaire assessing subjectively perceived ability to perform work. Includes a question assessing a participant’s current ability to perform any work compared to their lifetime best (peak form) on a scale of 0 to 10 (question 1 from the WAI Work Ability Index), and questions related to mental resources such as feelings of hope for the future, activity level, mood variability, and satisfaction with activities. The mental resources scale score was calculated as the sum of scores on individual subscales, each assessed on a 5-point scale where 0 means never and 4 means often (Pokorski, 1998). The reliability of the scale as measured by Cronbach's α coefficient in this study group was 0.86.

• Copenhagen Psychosocial Questionnaire (COPSOQ II) by Kristensen, Hannerz, Hogh and Borg, 2005, Polish adaptation by Baki Ł. — includes a self-efficacy subscale that describes generalized beliefs of participants that, regardless of circumstances, they are able to cope with difficult problems, unexpected situations, and realize their own plans and intentions. This subscale consists of 6 questions answered on a 4-point scale, where 1 means completely fitand 4 means not fit at all. According to the original test authors’ recommendations, raw scores of all COPSOQ II items should be transformed to a 0–100 scale (Baka, 2019). The reliability of the subscale as measured by Cronbach's α coefficient in this study group was 0.95.

• Rosenberg’s Self-Esteem Scale (SES), Polish adaptation by I. Dzwonkowska, K. Lachowicz-Tabaczek and M. Łaguna — a scale assessing general self-esteem, understood as a relatively stable disposition reflecting a positive or negative conscious attitude towards the Self. It includes 10 statements rated on a 4-point scale, where 1 means strongly agree and 4 means strongly disagree. When scoring, positively worded are reverse-coded so that higher scores correspond to higher self-esteem (Dzwonkowska, Lachowicz-Tabaczek, Łaguna, 2008). The reliability of the scale as measured by Cronbach's α coefficient in this study group was 0.76.

• Multidimensional Scale of Perceived Social Support (MSPSS) by G. Zimet, N. Dahlem, S. Zimet & G. Farley, Polish adaptation by K. Buszman and H. Przybyła-Basista — this scale captures the multidimensionality of perceived social support, recognizing three primary sources of support: a significant person, family, and friends. Participants respond on a 7-point scale, where 1 means strongly disagree and 7 means strongly agree (Buszman, Przybyła-Basista, 2017). The reliability of the overall scale was 0.78, and for individual subscales: significant person – 0.85, family – 0.84, friends – 0.85.
