# Supplementary material for: Factors influencing the participation of people with disabilities in digital skills training in Poland
Source: PLoS One. 2026 Jun 17;21(6):e0349514. doi: 10.1371/journal.pone.0349514 (PMC13274863; doi:10.1371/journal.pone.0349514)
Supplement: S1 Table — (DOCX) [file pone.0349514.s002.docx]

Supplementary files - S1 Table.

**S1 Table. Distribution of answers of respondents regarding self-assessment of selected 27 digital competences (in %, N=449).**

|  | **Lack of competencies** | **Low level of competencies** | **Average level of competencies** | **High level of competencies** | **Very high level of competencies** |
| --- | --- | --- | --- | --- | --- |
| **Edit and format texts in a computer program** | 15% | 25% | 31% | 19% | 10% |
| **Organizing files** | 11% | 24% | 31% | 21% | 13% |
| **Using Excel functions** | 17% | 25% | 35% | 15% | 8% |
| **Creating charts in Excel** | 26% | 30% | 20% | 16% | 8% |
| **Creating presentations in Power Point** | 26% | 22% | 27% | 16% | 9% |
| **Setting up an email account** | 10% | 22% | 28% | 23% | 16% |
| **Sending and receiving e-mails** | 6% | 16% | 26% | 25% | 28% |
| **saving photos, documents, etc.** | 7% | 18% | 28% | 24% | 23% |
| **Ensure digital security** | 13% | 30% | 30% | 19% | 8% |
| **Using new media** | 12% | 25% | 32% | 18% | 13% |
| **Managing platforms and data** | 15% | 32% | 29% | 18% | 6% |
| **Viewing data, information and digital content** | 10% | 24% | 30% | 24% | 12% |
| **Searching for data, information and digital content** | 8% | 25% | 29% | 24% | 13% |
| **Filtering data, information and digital content** | 13% | 25% | 31% | 19% | 12% |
| **Managing automation** | 27% | 30% | 27% | 12% | 4% |
| **Managing data, information and digital content** | 20% | 28% | 30% | 16% | 5% |
| **Communicating using digital technologies** | 15% | 25% | 36% | 16% | 8% |
| **Collaboration using digital technologies** | 20% | 24% | 33% | 18% | 4% |
| **Evaluating data, information and digital content** | 18% | 30% | 31% | 16% | 5% |
| **Creating digital content** | 26% | 31% | 25% | 12% | 5% |
| **Programming** | 50% | 35% | 10% | 3% | 2% |
| **Sharing content through digital technologies** | 23% | 28% | 27% | 17% | 7% |
| **Selling in the digital economy** | 29% | 32% | 26% | 10% | 4% |
| **Management of virtual teams** | 36% | 34% | 22% | 6% | 2% |
| **Sharing information and resources using digital technologies** | 27% | 29% | 27% | 12% | 5% |
| **Knowledge of device protection measures, ability to restrict access to data** | 27% | 25% | 30% | 13% | 5% |
| **Managing digital identities** | 29% | 26% | 28% | 12% | 5% |
