# Supplementary material for: Factors influencing the participation of people with disabilities in digital skills training in Poland
Source: PLoS One. 2026 Jun 17;21(6):e0349514. doi: 10.1371/journal.pone.0349514 (PMC13274863; doi:10.1371/journal.pone.0349514)
Supplement: S2 Table — (DOCX) [file pone.0349514.s003.docx]

Supplementary files – S2 Table.

**S2 Table. Characteristics of participants with disabilities who did and did not take part in digital competence training – Results of Mann-Whitney U test and Chi-square test (N = 449)**

| Variable | Persons participating in digital competence training | Persons does not participating in digital competence training | U Manna-Whitney’a test  Chi square test |
| --- | --- | --- | --- |
| *Age* | 38,8 lat | 43,6 lat | U=20294,00  P<0,001 |
| *Level of disability*  Mild  Moderate  Severe | 45,3  44,8  9,9 | 30,4  50,7  18,9 | ꭓ^2^=13,640  P=0,001 |
| *Current workability compared to lifetime best* | 6,7 | 5,1 | U=33334,50  P<0,001 |
| *Mental rsoures* | 8,3 | 6,3 | U=35260,50  P<0,001 |
| *Social supprot* | 61,3 | 55,5 | U=2999700  P<0,001 |
| *Significant person* | 20,4 | 18,9 | U=28661,00  P=0,011 |
| *Family* | 20,6 | 18,4 | U=30318,50  P<0,001 |
| *Friends* | 20,3 | 18,2 | U=29906,00  P<0,001 |
| *Self-esteem* | 29,1 | 26,8 | U=30502,500  P<0,001 |
| *Self-efficacy* | 58,7 | 44,3 | U=33062,00  P<0,001 |
| *Employment/no employment* | 72,4% zatrudnionych | 43,3% zatrudnionych | ꭓ^2^=39,058  P<0,001 |
| *Participation in any courses*  Yes  No | 100%  0% | 91,2%  8,8% | ꭓ^2^=378,675  P<0,001 |
| *Attending trainings at any time*  Yes  No | 100%  0% | 53,5%  46,5% | ꭓ^2^=139,321  P<0,001 |
| *It is essential to have and use digital competences in the enterprise*  *Yes*  *No* | 66%  34% | 37%  63% | ꭓ^2^=60,843  P<0,001 |
| *Using new technologies in everyday life*  Yes  No | 100%  0% | 91,7%  8,3% | ꭓ^2^=20,048  P<0,001 |
| *Using assistive technologies in everyday life*  Yes  No | 52,36%  47,4% | 35%  65% | ꭓ^2^=14,030  P<0,001 |
| *The belief that digital competences are important employee competences*  *Yes*  *No*  *I have no opinion* | 84%  6%  10% | 55%  12%  33% | ꭓ^2^=44,992  P<0,001 |
| *The belief that modern employees should have advanced digital competences*  Yes, this is the direction in which employees should develop and the competences they should acquire. Their basic level is not enough in the modern labour market.  It depends on the profile of the company in which they are employed  No, there is no need for them to have competences at an advanced level. Basic skills in this area are enough. | 53%  41%  6% | 27%  54%  19% | ꭓ^2^=41,186  P<0,001 |
| *Interest in having digital competence at an advanced level*  *Yes*  *No*  *I do not know* | 71%  21%  8% | 31%  35%  34% | ꭓ^2^=75,835  P<0,001 |
| *Ability to identify digital competence gaps*  Definitely yes  Rather yes  Rather no  Definitely no  I do not know | 14,2%  36,2%  19%  5,2%  25,4% | 6,5%  32,7%  19,4%  12,4%  29% | ꭓ^2^=14,233  P=0,007 |
| *Directing to digital competences training by employers*  *Yes*  *No, I attend them on my own* | 44%  56% | 34%  66% | ꭓ^2^=230,137  P<0,001 |
| *Assessment of the availability of digital competence training for people with disabilities*  Yes  Only some elements  No  I do not know | 50%  34,9%  13,8%  1,3% | 18%  17%  11%  54% | ꭓ^2^=270,590  P<0,001 |
| *Future plans for the development of digital competences (they want to use such training)*  *Yes*  *No*  *I do not know* | 64%  13%  23% | 21%  20%  59% | ꭓ^2^=87,738  P<0,001 |
