# Supplementary material for: Factors influencing the participation of people with disabilities in digital skills training in Poland
Source: PLoS One. 2026 Jun 17;21(6):e0349514. doi: 10.1371/journal.pone.0349514 (PMC13274863; doi:10.1371/journal.pone.0349514)
Supplement: S3 Table — (DOCX) [file pone.0349514.s004.docx]

Supplementary files – S3 Table.

**S3 Table. VIF test results (Variance Inflation Factor)**

|  | B | Standard Error | Beta | t | Significance | Tolerance | VIF |
| --- | --- | --- | --- | --- | --- | --- | --- |
| (Constant) | 1,535 | 0,196 |  | 7,823 | <,001 |  |  |
| Gender | -0,009 | 0,033 | -0,009 | -0,284 | 0,776 | 0,895 | 1,118 |
| Age | -0,002 | 0,001 | -0,063 | -1,794 | 0,074 | 0,8 | 1,249 |
| Place of residence | 0,013 | 0,015 | 0,03 | 0,892 | 0,373 | 0,888 | 1,126 |
| Level of education | -0,015 | 0,01 | -0,058 | -1,486 | 0,138 | 0,645 | 1,55 |
| Degree of disability | -0,049 | 0,027 | -0,066 | -1,83 | 0,068 | 0,741 | 1,349 |
| Type of disability | -0,003 | 0,021 | -0,004 | -0,133 | 0,895 | 0,911 | 1,097 |
| Duration of disability | 0,001 | 0,001 | 0,017 | 0,482 | 0,63 | 0,822 | 1,216 |
| Everyday use of new technologies | -0,121 | 0,085 | -0,048 | -1,427 | 0,154 | 0,881 | 1,135 |
| Everyday use of assistive technologies | -0,039 | 0,036 | -0,039 | -1,085 | 0,279 | 0,757 | 1,322 |
| Assessment of self-reported digital competences | 0,043 | 0,025 | 0,073 | 1,755 | 0,08 | 0,569 | 1,759 |
| Interest in developing digitalcompetences | -0,048 | 0,025 | -0,076 | -1,958 | 0,051 | 0,648 | 1,543 |
| Belief in the importance of advanced digital competences for modern employees | -0,024 | 0,027 | -0,032 | -0,89 | 0,374 | 0,732 | 1,365 |
| Belife in the accessibility of digital competence training for people with disabilities | -0,199 | 0,013 | -0,623 | -15,144 | <,001 | 0,578 | 1,73 |
| Willingnes to use digital competence training in the future | -0,04 | 0,021 | -0,074 | -1,899 | 0,058 | 0,641 | 1,56 |
| Assessment of current ability to work compared to the best ability to work in life (WAI1) | -0,009 | 0,007 | -0,049 | -1,213 | 0,226 | 0,605 | 1,652 |
| Mental resources (WAI) | 0,009 | 0,009 | 0,048 | 1,008 | 0,314 | 0,439 | 2,277 |
| Self-efficacy (COPSOQ) | -0,001 | 0,001 | -0,028 | -0,658 | 0,511 | 0,559 | 1,787 |
| Social support (MSPSS) | -0,001 | 0,001 | -0,032 | -0,858 | 0,391 | 0,705 | 1,418 |
| Self-assessment (SES) | 0,003 | 0,003 | 0,035 | 0,897 | 0,37 | 0,642 | 1,558 |
